# Recent advances in acute promyelocytic leukaemia

**DOI:** 10.12688/f1000research.10736.1

**Published:** 2017-07-28

**Authors:** Chin-Hin Ng, Wee-Joo Chng

**Affiliations:** 1 National University Cancer Institute, Singapore, Singapore

**Keywords:** leukemia, PML, RARA

## Abstract

Acute promyelocytic leukaemia (APML) is a subtype of leukaemia arising from a distinct reciprocal translocation involving chromosomes 15 and 17, which results in the *PML-RARA* fusion gene. Over the past three decades, APML has been transformed from a highly fatal disease to a highly curable one. This drastic improvement is because of the introduction of a new treatment strategy with all-trans retinoic acid and, more recently, arsenic trioxide. The revolutionary treatment of APML has also paved the way for a new cancer treatment, which is genetically targeted therapy. In this review, we look into this amazing journey of transformation and provide recent advances in the management of APML.

## Introduction

Acute myeloid leukaemia (AML) is a heterogeneous disease that results from the abnormal maturation of myeloid lineages. Based on the early French-American-British (FAB) classification, acute promyelocytic leukaemia (APML) is one of the subtypes (M3) of AML. It arises from a distinct reciprocal translocation involving chromosomes 15 and 17, which results in the *PML-RARA* fusion gene, t(15;17)(q24.1;q21.1) ^1^. A number of rarer variant translocations involving the *RARA* gene associated with APML, including t(11;17) and t(5;17), have also been identified ^2^. The disruption of the *RARA* gene results in maturation arrest of myeloid progenitors at the promyelocytic stage, and treatment with all-trans retinoic acid (ATRA) was able to induce differentiation in *in vitro* and *in vivo* models ^3^ and induce complete remission (CR) in patients with APML, though with short duration of CR as monotherapy ^4^. Subsequent incorporation of ATRA into standard chemotherapy (CT) in many studies and, more recently, the introduction of arsenic trioxide (ATO) into the treatment strategy demonstrated dramatic improvement in clinical outcomes, transforming a previously deadly disease into a highly curable one ^5^. However, a few issues remain unsettled; these include the long-term safety profile and efficacy of ATO, the need for maintenance therapy in the era of ATRA/ATO, and the effectiveness of differentiation syndrome (DS) prophylaxis to name a few.

## Variant translocation in acute promyelocytic leukaemia

Translocation involving *PML* and *RARA* genes, accounting for more than 90% of cases, is by far the most common fusion partner in APML. Other fusion partners include *PZLF-RARA* ^6^, *NPM-RARA* ^7^, *NuMA-RARA* ^8^, and the more recently discovered *STAT5b-RARA* ^9^ and *BCOR-RARA* ^10^. These rarer fusion genes account for around 5% of the total. The clinical significance of these variant fusion genes is their response to ATRA. *PZLF-RARA* and *STAT5b-RARA* cases were refractory to ATRA, whereas *NPM-RARA* and *NuMA-RARA* were reported to be responsive. *BCOR-RARA* was also responsive to ATRA but carries a higher risk of relapse ^[Bibr ref-10], 11^.

## *FLT3-*ITD in acute promyelocytic leukaemia

FMS-like tyrosine kinase 3 (FLT3) is a transmembrane tyrosine kinase receptor that is involved in cell proliferation. Mutation in the *FLT3* gene that results in internal tandem duplication (ITD) and constitutively active tyrosine kinase receptor is relatively common in AML, especially in those with a normal karyotype. AML with *FLT3*-ITD is associated with a high risk of relapse and generally is considered to be a high-risk disease requiring allogeneic stem cell transplantation. However, the clinical impact of *FLT3*-ITD in APML is still controversial.

The incidence of *FLT3*-ITD in APML is 20 to 30% ^12, [Bibr ref-13]^. According to the International Consortium on APML Study, *FLT3*-ITD was associated with a significantly higher presenting white blood cell (WBC) count and hence with high-risk groups. With a median follow-up of 38 months, *FLT3*-ITD APML showed an inferior overall survival (OS) at 3 years (62% versus 82%). In the multivariate analysis that included age, presenting WBC count, albumin, and *FLT3*-ITD status, *FLT3*-ITD-positive APML was independently associated with inferior OS. However, the cut-off used for the WBC count was 50 × 10 ^9^/L instead of the more widely used 10 × 10 ^9^/L ^12^. In a separate study involving 245 APML cases, *FLT3*-ITD status did not seem to affect clinical outcomes, especially in those patients who received ATO ^13^. Similarly, in an Italian series of 159 APML cases, *FLT3*-ITD-positive APML had no significant impact on either event-free survival (EFS) or cumulative incidence of relapse in patients receiving ATRA-ATO. However, there was a trend towards inferior EFS in *FLT3*-ITD cases receiving the ATRA plus CT (ATRA-CT) regimen ^14^.

A meta-analysis involving 11 studies and 1,063 patients, who mostly received the ATRA-CT regimen, showed an inferior OS and disease-free survival (DFS) in *FLT3*-ITD-positive APML ^15^.

The clinical impact of *FLT3*-ITD is likely muddled by the concurrent high WBC count, which is the most important risk factor in APML. Hence, the role of *FLT3*-ITD in further risk stratification of the currently available method is likely redundant ( Table 1). The prognostic impact of *FLT3*-ITD in the era of ATRA-ATO upfront treatment might not be significant after all. Furthermore, the use of an FLT3 inhibitor in APML has yet to be established. Routine checking of *FLT3*-ITD status is probably unwarranted.

**Table 1.** Risk stratification in acute promyelocytic leukaemia ^67^.

Risk	White blood cells, × 10 ^9^/L	Platelets, × 10 ^9^/L	Three-year relapse- free survival
Low	<10	>40	98%
Intermediate	<10	<40	89%
High	>10	-	70%

## Other prognostic factors

In a recent review by Testa and Lo-Coco, immunophenotypes in APML might play some roles in prognostication and are associated with characteristic clinical phenotypes ^16^. CD56 is over-expressed in about 10% of APML and often is associated with high WBC count ^14^. Its expression is associated with increased relapse risk and inferior relapse-free survival (RFS), EFS, and OS in a number of studies ^17– 19^. CD2 expression was also found in around 24% of APML cases and was associated with CD34 positivity, higher WBC count, and microgranular subtype ^20, 21^. In a separate series of 132 Chinese patients with APML, CD2 expression was found to be associated with early death. However, in the multivariate analysis involving CD2, CD34, CD56, and WBC count, only increased WBC count was an independent prognostic factor for early death and OS ^22^. In an analysis of 114 APML cases, Breccia *et al.* observed that the CD34/CD2-positive subgroup displayed several differential properties—including higher frequencies of M3v (27% versus 7%), bcr3 transcript subtype (73% versus 32%), higher incidence of DS (26% versus 12%), higher rate of relapse (66% versus 17%), and lower OS (88% versus 95%)—compared with CD34-negative patients ^23^.

Additional mutations are not uncommon in APML, particularly in the high-risk group. Patients with mutations of the epigenetic modifier genes were reported to have a significantly reduced OS and RFS compared with patients lacking these mutations ^24^.

## Additional chromosomal abnormalities in acute promyelocytic leukaemia

In a large UK Medical Research Council (MRC) trial with more than 5,000 patients with AML, the cytogenetics of 607 patients with APML were further analysed to elucidate the clinical impact of additional chromosomal abnormalities ^25^. The patients with APML received ATRA and anthracycline-based CT, and the presence of additional chromosomal abnormalities did not affect the clinical outcomes. This included patients with 17p or other adverse karyotypes known to confer poor outcome in AML.

## Mutational landscape in acute promyelocytic leukaemia

In a recently published study on the mutational analysis of APML by Madan *et al.* from the Cancer Science Institute of Singapore, a comprehensive mutational landscape of primary and relapsed APML was unveiled ^26^; 153 primary and 69 relapsed APML samples were explored by using whole-genome sequencing (n = 12) and subsequent targeted sequencing of 368 genes. *FLT3* emerged as the most frequently altered gene in primary APML samples. This included 27% of cases with *FLT3*-ITD (45 out of 165 initial diagnosis samples) and 16% of cases (26 mutations in 25 samples) with point mutations of *FLT*. Other commonly observed mutated genes include *WT1* (14%), *NRAS* (10%), *KRAS* (4%), *ETV6* (1%), and *EZH2* (1%). Interestingly, somatic mutations in known leukaemia-related genes (for example, *DNMT3A*, *NPM1*, *TET2*, *IDH1*, *IDH2*, and *ASXL1*) were absent or rarely observed in APML.

## Thrombosis in acute promyelocytic leukaemia

Whereas bleeding complications are common in APML, thrombotic events occurring before and during therapy are not uncommon. The incidence of thrombotic events appeared to be higher in patients who received ATRA compared with those in the pre-ATRA period ^27^. The largest study reported an incidence of 5% (39 out of 739) in patients who received ATRA-CT ^28^. The incidence increased to 9% in patients who received ATRA and tranexamic acid ^17^. Rashidi *et al.* conducted a careful review on the reported cases of thrombotic events in APML treated with an ATRA-containing regime ^29^. In the 94 cases collated from the literature review, it was observed that more than 80% of such events occurred before or during induction therapy and that deep vein thrombosis/pulmonary embolism, cardiac events, and cerebrovascular accidents constituted more than 75% of all cases. The incidence was fairly similar between those occurring before and during treatment with ATRA, possibly suggesting that ATRA therapy would not alter the thrombotic risk in APML. The underlying pathophysiology of thrombosis in this small subgroup of APML is still unclear. Treatment-related factors such as the use of ATRA, tranexamic acid, or even a chemotherapeutic agent might have increased the risk of thrombosis ^18, 19, 30^. Disease-related factors such as a higher WBC count, type of PML/RARa transcript, *FLT3*/ITD mutation, and positivity for CD2 or CD15 were reported to be associated with thrombosis in a large study of patients who received ATRA, CT, and prophylactic tranexamic acid ^17^.

## Update on acute promyelocytic leukaemia treatment

Since the introduction of ATRA into the treatment of APML slightly more than three decades ago, the clinical outcome of this deadly disease has changed completely ^5^. In the pre-ATRA era, anthracycline-based CT was the standard of treatment and reported CR rates were 75 to 80% ^31, 32^. However, the median duration of remission was only 11 to 25 months, and only 35 to 45% were cured by CT alone. Many subsequent clinical trials that incorporated ATRA into anthracycline-based CT have further improved the CR rates to 90 to 95% and DFS to around 70% or more ( Table 2).

**Table 2.** Outcome in patients with acute promyelocytic leukaemia treated with all-trans retinoic acid-based regimens.

Reference group	Number	Complete remission, %	Disease-free survival, %	Overall survival, %
Tallman *et al.* ^61^	350	ATRA: 70 CT: 73	ATRA: 69 (5 years) CT: 29 (5 years)	ATRA: 69 (5 years) CT: 45 (5 years)
Fenaux *et al.* ^68^	ATRA: 54 CT: 47	ATRA: 91 CT: 81	EFS at 12 months ATRA: 79 CT: 50	
Avvisati *et al.* ^69^	807	94.3	EFS: 68.9 (12 years)	76.5 (12 years)
Iland *et al.* ^53^	101	90	71 (4 years)	84 (4 years)
Sanz *et al.* ^70^	560	91	84 (5 years)	82 (5 years)
Asou *et al.* ^71^	283	94	68.5 (6 years)	83.9 (6 years)

ATRA, all-trans retinoic acid; CT, chemotherapy; EFS, event-free survival.

## Moving from chemotherapy-based to chemotherapy-free treatment

The use of ATO in the treatment of APML was first reported by a Chinese group from Harbin Medical University in 1996 ^33^. Thirty newly diagnosed and 42 relapsed APML patients received single-agent ATO. CR rates of 73% and 52%, respectively, were observed. Another group, from the Shanghai Institute of Hematology, has reported high CR rates in newly diagnosed APML and relapsed cases (85.7% and 83.9%, respectively), further confirming the efficacy of ATO monotherapy ^34^. Interestingly, ATO monotherapy did not result in significant myelosuppression in these early cohorts. These early observations have led to a plethora of case series and clinical trials internationally, subsequently establishing ATO as the second line of treatment ^35– 39^. As a single agent, ATO is capable of inducing CR in 85 to 90% of relapsed cases. Besides having a high CR rate, ATO is capable of penetrating the blood–brain barrier, making it an ideal salvage treatment because central nervous system diseases have been reported in approximately 10% of relapsed APML ^40– 42^. Upfront monotherapy with ATO has been performed since the early days of ATO use, and the results of long-term follow-up of these case series have been reported. A case series with elderly APML patients who were not fit for CT has shown a durable remission and results comparable to those of standard ATRA-CT in younger patients ^43^. The 10-year cumulative incidence of relapse was 10.3%, which compared favourably to ATRA-CT. In a separate study, single-agent ATO in patients with newly diagnosed APML provided a 5-year DFS of 80%, and minimal short-term toxicity and no long-term toxicity have been observed thus far ^44, 45^.

Early pre-clinical studies demonstrated synergistic activity when ATRA was combined with ATO in mouse studies ^34, 35^. With the long-term safety and efficacy data available for ATO, it is rational to combine ATO with ATRA in upfront treatment for APML. The group from the Shanghai Institute of Hematology was the first to report the use of this combination, in a cohort of 85 patients with newly diagnosed APML ^46, 47^. The group reported a high CR rate (94.1%) and a 5-year EFS and OS of 89.2% and 91.7%, respectively. The toxicities were mild and reversible. The group has also found that poor prognostic factors such as high WBC count (>10 × 10 ^9^/L) did not influence the outcome. Subsequently, more groups have reported the use of ATRA/ATO in newly diagnosed patients ( Table 3) ^38, 47– 52^.

**Table 3.** Summary of the studies using all-trans retinoic acid/arsenic trioxide as upfront treatment.

References	Year	Number	Complete remission rate	Event-free survival	Overall survival	Remarks
Estey *et al.* ^38^	2006	Low risk: 25 High risk: 19	Low risk: 96% High risk: 79%			Non-randomised. Median follow-up of 16 months. No relapse in the low-risk group. Three relapsed in the high-risk group. Fifteen high-risk patients received an additional single dose of GO 9 mg/m ^2^ on day 1, and three had additional idarubicin chemotherapy.
Ravandi *et al.* ^48^	2008	Low risk: 56 High risk: 26	Low risk: 95% High risk: 81%	3-year EFS Low risk: 89.2% High risk: 64.6%	3-year OS Low risk: 89.2% High risk: 75.4%	Non-randomised. Median follow-up of 99 weeks. Included all risk groups. High-risk patients received an additional dose of GO 9 mg/m ^2^ on day 1, and those who developed leukocytosis during treatment in the low-/intermediate-risk group received additional GO as well.
Hu *et al.* ^47^	2009	85	94.10%	5-year EFS: 89.2% 5-year RFS: 94.8%	5-year OS: 91.7%	Non-randomised. Included all risk groups.
Lo-Coco *et al.* ^49^	2013	ATRA-ATO: 77 ATRA-CT: 79	ATRA-ATO: 100% ATRA-CT: 95%	2-year EFS ATRA-ATO: 97% ATRA-CT: 86%	2-year OS ATRA-ATO: 99% ATRA-CT: 91%	Randomised. Included only low- and intermediate-risk patients. Median follow-up of 34.4 months.
Burnett *et al.* ^50^	2015	ATRA-ATO: 116 ATRA-CT: 119	ATRA-ATO: 94% ATRA-CT: 89%	4-year EFS ATRA-ATO: 91% ATRA-CT: 70%	4-year OS ATRA-ATO: 93% ATRA-CT: 89%	Randomised. Included all risk groups. Low/intermediate risk, n = 178; high risk, n = 57. High-risk group received one dose of GO 6 mg/m ^2^ during induction.
Abaza *et al.* ^51^	2016	Low risk: 133 High risk: 54	Low risk: 95.5% High risk: 96.3%	5-year EFS Low risk: 87% High risk: 81%	5-year OS Low risk: 89% High risk: 86%	Non-randomised. Included all risk groups. High-risk patients received an additional dose of GO 9 mg/m ^2^ on day 1, and those who developed leukocytosis during treatment in the low-/intermediate-risk group received additional GO as well.
Platzbecker *et al.* ^52^	2016	ATRA-ATO: 127 ATRA-CT: 136	ATRA-ATO: 100% ATRA-CT: 97%	4-year EFS ATRA-ATO: 97.3% ATRA-CT: 80.0%	4-year OS ATRA-ATO: 99.2% ATRA-CT: 92.6%	Randomised. Included only the low-/intermediate-risk group.

ATRA-ATO, all-trans retinoic acid plus arsenic trioxide; ATRA-CT, all-trans retinoic acid plus chemotherapy; EFS, event-free survival; GO, gemtuzumab ozogamicin; OS, overall survival.

The final report of the randomised Italian-German APL0406 trial was recently published, confirming the efficacy and safety of ATRA/ATO, especially in the low-risk group ^52^. This was an expanded cohort of the randomised controlled trial reported in 2013 by the GIMEMA-SAL-AMLSG study groups ^49^. In total, 263 patients with newly diagnosed non-high-risk APML were accrued and randomly assigned to ATRA-ATO or ATRA-CT in a 1:1 ratio. CR rates of 100% and 97% were reported in the ATRA-ATO and ATRA-CT arms, respectively ( *p* = 0.12). Significant superior EFS, cumulative incidence of relapse, and OS at 50 months were observed in the ATRA-ATO arm (97.3% versus 80%, *p* = 0.001; 1.9% versus 13.9%, *p* = 0.0013; and 99.2% versus 92.6%, *p* = 0.0073, respectively). Of note, induction death occurred only in the ATRA-CT arm (four patients, or 3%).

The MD Anderson group has also updated their expanded cohort of 187 patients with a median follow-up of 47.6 months ^51^. It was a non-randomised trial using ATO-ATRA upfront in all risk groups. However, the high-risk group was allocated to receive an additional gemtuzumab ozogamicin (GO) dose on day 1 of induction. Those who developed leukocytosis in low or intermediate risk during ATRA-ATO therapy were also given a dose of GO to control rising WBC count. Forty-five patients in the high-risk group received GO, and seven others received idarubicin (when GO was not available). In the low-risk group, 60 patients received cytoreduction therapy for leukocytosis. Fifty-one patients received GO and the remaining nine received idarubicin for cytoreduction. In total, 59.9% of the patients received cytoreductive therapy. The study recorded high CR rates in both low- and high-risk groups and no significant difference in EFS and OS between the risk groups.

MRC UK has also recently reported a large cohort of patients with newly diagnosed APML receiving ATRA-ATO as upfront treatment ^43, 50^. It was a randomised study and formed part of the AML17 study. In total, 235 patients were enrolled from 81 centres in the UK. The comparative arm received ATRA plus idarubicin as induction followed by ATRA-CT consolidation. It is important to note that the ATO dose and schedule were rather different from those reported in other studies. A higher ATO dose was given during the first week of induction but for only 5 days. In subsequent weeks, a higher dosage (0.25 mg/m ^2^) was given again twice weekly. However, the total ATO dose for the whole induction and the consolidation cycles was significantly higher in the AML17 trial compared with other groups ( Table 4). Twenty-eight (93%) of 30 high-risk patients in the ATRA-ATO arm received GO (the remaining two high-risk patients did not because there was no GO available on-site, and anthracycline was given instead). Furthermore, seven low-risk patients were also given GO for their rising WBC count. The strategy of using GO in the high-risk group and rising WBC count in the low-risk group was similar to that reported by the MD Anderson group. A high CR rate of 94% was documented in the ATRA-ATO arm compared with 89% in the ATRA-CT arm but was not statistically significant. The 4-year EFS was significantly better in the ATRA-ATO arm than in the ATRA-CT arm (91% versus 70%, *p* = 0.002). This significant benefit was apparent in low-risk patients—4-year EFS of 92% (95% confidence interval [CI] 84–97%) in the ATRA-ATO arm versus ** 71% in the ATRA-CT arm; hazard ratio (HR) 0.34 (0.15–0.75), *p* = 0.008—but not in the high-risk groups. The 4-year EFS and OS of this cohort appeared to be comparable to those of other groups (92% and 95%, respectively). This opens up the possibility of an alternative dosing schedule for the ATRA-ATO regimen, which is more pragmatic.

**Table 4.** Comparing the arsenic trioxide dosing between AML17 and APL0406 trials.

	AML17 trial	APL0406 trial
Induction cycle	Week 1: 0.3 mg/kg (day 1–5) Week 2–8: 0.25 mg/kg twice weekly	0.15 mg/kg per day (up to 60 days)
Consolidation (four cycles)	Week 1: 0.3 mg/kg (day 1–5) Week 2–4: 0.25 mg/kg twice weekly	0.15 mg/kg 5 days weekly for 4 weeks
Total arsenic trioxide dose	25 mg/kg	21 mg/kg

The superiority of ATRA-ATO over the conventional ATRA-CT regimen is undisputable in the low- and intermediate-risk group but not in the high-risk group. The addition of GO to the ATRA-ATO regimen in the high-risk group in most recently published studies has muddled the true efficacy of ATRA-ATO in this risk group. Although the cohort from Hu *et al.* did shed some light on the potential efficacy of ATRA-ATO in the high-risk group, the result came from a small and non-randomly assigned cohort ^47^. GO is currently still not available outside the clinical trial setting, hence using ATRA-ATO alone for the high-risk group is not recommended. More data on the use of ATRA-ATO in the high-risk group are needed to support this recommendation. Nevertheless, in the APL4 trial by the Australia–New Zealand group, idarubicin was incorporated into the ATRA-ATO regimen during induction followed by ATRA-ATO consolidation therapy in all risk groups. Among the 124 patients, four induction deaths were reported and two patients withdrew consent. The remaining 118 patients achieved haematologic CR ^53^.

## Paediatric acute promyelocytic leukaemia

The incidence of APML in children is much lower compared with adults. It constitutes only 5 to 7% of AML in children ^20– 22^. The optimal therapy is still not established, but the increasing use of ATRA-CT and the ATRA-ATO regimen has largely improved the outcome ^54^. Adult protocols were often applied to paediatric APML. The outcomes appeared to be comparable to those of the adult population ^22^. Nevertheless, there is concern over the long-term side effects, particularly with ATO. In a small series reported by a German group, 11 children with low-risk APML received ATRA-ATO and all developed hyperleukocytosis. Two patients experienced reversible severe side effects. One developed osteonecroses at both femurs, seizures, and posterior reversible encephalopathy syndrome, and the other patient had an abducens paresis ^55^. In contrast, two Chinese groups reported an excellent long-term outcome with minimal chronic toxicities in patients who received ATO ^24, 56, 57^.

## Incidence of differentiation syndrome and the effectiveness of prophylaxis

DS is a life-threatening condition associated more commonly with ATRA- and ATO-based treatment, although DS could occur without ATRA/ATO. It is characterised by fever, peripheral oedema, pulmonary infiltrates, hypoxemia, respiratory distress, hypotension, renal and hepatic dysfunction, and serositis resulting in pleural and pericardial effusions. Rates of incidence of DS in the ATRA-CT era were reported to be around 25% in the large series PETHEMA LPA96 and LPA99 trials ^28^ and 30% in ATO monotherapy in the newly diagnosed or relapsed setting ^58, 59^; a more recent ATRA-ATO regimen reported incidences of between 15 and 20% with steroid prophylaxis ^48, 49^. The use of steroid was based on observations in the LPA96 and LPA99 trials, where patients in LPA99 were given prednisolone prophylaxis from the start whereas LPA96 used selective intravenous dexamethasone prophylaxis in patients with a WBC count of more than 5 × 10 ^9^/L. The incidence of severe DS was significantly higher in the latter. In a combined analysis of the two trials, presenting WBC count of more than 5 × 10 ^9^/L was an independent predictor of severe DS ^28^ In a separate study, those with DS were found to have higher WBC count peaks compared with those without DS. However, some patients who developed leukocytosis during the treatment never progressed to DS ^60^. More recently, the large UK MRC AML17 trial reported an incidence of 21% in the ATRA-ATO arm without the use of steroid prophylaxis, although those who developed leukocytosis were given an additional dose of GO ^50^. The rate was close to those reported in the studies with steroid prophylaxis. This raised the question of the effectiveness of prophylactic steroids to prevent DS. Although there has been no direct evidence to support the routine use of steroid prophylaxis, its use in patients with high presenting WBC count is appropriate. Timely administration of cytoreductive therapy such as cytarabine in patients with rising WBC count during treatment with an ATRA/ATO-containing regimen would reduce the risk of DS development, even though this was also based on indirect evidence.

## Do we still need maintenance therapy in the era of all-trans retinoic acid/arsenic trioxide?

Maintenance therapy was originally part of the design of the ATRA-CT regimen, and a subsequent randomisation trial confirmed the benefit of such therapy ^61, 62^. However, in the era of ATRA-ATO, the role of maintenance has come into question. The Intergroup APL0406 randomised phase III trial, which did not include maintenance therapy for those who received ATRA plus ATO, has demonstrated the non-inferiority of ATRA plus ATO compared with ATRA plus idarubicin (AIDA) in low- or intermediate-risk APL ^52^. In the UK MRC trial, the absence of maintenance therapy in the ATRA-ATO group similarly confirmed the non-inferiority of ATRA plus ATO compared with AIDA in all risk groups ^50^. In a non-inferiority study, 105 adults with newly diagnosed low- or intermediate-risk APL underwent induction with ATRA-CT followed by consolidation that included ATO ^63^. The 68 patients who achieved complete molecular response were randomly assigned to observation alone or maintenance therapy with ATRA, 6-mercaptopurine (6-MP), and methotrexate. After a median follow-up of 36 months, no patients who achieved complete molecular response have relapsed.

In a recent study reported by the Japan Adult Leukaemia Study Group, tamibarotene, a synthetic retinoic acid, might improve the RFS in high-risk APML ^64^. In this phase III study, 269 patients who achieved CR after ATRA-CT induction and consolidation cycles were randomly assigned to either ATRA or tamibarotene maintenance. The RFS rates at 4 years were 84% for the ATRA arm and 91% for the tamibarotene arm (HR 0.54, 95% CI 0.26 to 1.13). However, when the analysis was confined to 52 high-risk patients, the difference was statistically significant with 4-year RFS rates of 58% for the ATRA arm and 87% for the tamibarotene arm (HR 0.26, 95% CI 0.07 to 0.95).

In the low-/intermediate-risk group where ATRA-ATO has now become the standard of treatment, conventional maintenance therapy can be safely omitted. In the high-risk group, the omission of maintenance can be considered if an ATRA–ATO-containing regimen is used in conjunction with another cytotoxic agent such as idarubicin and has achieved molecular remission at the end of consolidation.

## Moving towards total oral therapy in the near future

Zhu *et al.* has recently reported the use of oral arsenic in combination with ATRA and showed a non-inferiority outcome when compared with intravenous ATO plus ATRA ^65^. In total, 242 patients with APML were randomly assigned in a 1:1 fashion to oral tetra-arsenic tetra-sulphide (As4S4) (60 mg/kg) or ATO (0.16 mg/kg) combined with ATRA during induction therapy. After achieving CR, all patients received three further courses of consolidation CT followed by maintenance therapy with sequential ATRA followed by As4S4 or ATO for 2 years. With a median follow-up time of 39 months, DFS rates at 2 years were 98.1% (106 out of 108) in the As4S4 group and 95.5% (107 out of 112) in the ATO group. The CR rate was not significantly different between the two groups (As4S4 versus ATO, 99.1% versus 97.2%, respectively; *p* = 0.62). The OS rates at 3 years were 99.1% for As4S4 and 96.6% for ATO ( *p* = 0.18). The rates of adverse events were similar. In a later study conducted by the same group, Zhu *et al.* demonstrated the feasibility of out-of-hospital treatment using oral As4S4 and ATRA without CT ^66^. In total, 20 consecutive non-high-risk APML patients were enrolled in this pilot study. Complete molecular remission was achieved in all patients. Post-remission therapy included oral As4S4 on a schedule of 4 weeks on/4 weeks off and ATRA on a schedule of 2 weeks on/2 weeks off for 7 months. At the median follow-up of 14 months (range of 8 to 19 months), no patient had molecular relapse. DS occurred in two patients and grade 3 or 4 adverse liver toxicities were observed in three patients. A prospective multicentre, randomised trial comparing oral As4S4 and ATRA with ATO and ATRA is ongoing in China.

## Conclusions

Similar to the success story of CML, the amazing journey of treatment transformation in APML has been a rare success story in cancer treatment. Both have paved the way in which cancer should be treated—molecularly targeted, total oral administration, highly effective, and yet with minimal toxicity.
